# The Design of a Fiber-Coupling Micro-Lens Array for an M × N Wavelength-Selective Switch

**DOI:** 10.3390/mi15030307

**Published:** 2024-02-23

**Authors:** Jiaqi Hao, Yunshu Gao, Chengcheng Dong, Zeyuan Meng, Genxiang Chen

**Affiliations:** 1 College of Science, Minzu University of China, Beijing 100081, China; hd_132500@163.com (J.H.); dcc199907@163.com (C.D.); mzyzzty@163.com (Z.M.); gxchen_bjtu@163.com (G.C.); 2 Engineering Research Center of Photonic Design Software, Ministry of Education, Beijing 100081, China

**Keywords:** wavelength-selective switch, fiber-coupling micro-lens, VirtualLab Fusion

## Abstract

The M × N port wavelength-selective switch (WSS) is a crucial device used for Reconfigurable Optical Add/Drop Multiplexors and optical switching nodes in optical communication systems. The primary function of an M × N port WSS is to simultaneously transmit and switch multiple input optical signals from input fiber ports to output fiber ports through spatial light coupling. The port array module in a WSS that is responsible for coupling the spatial beam with the fiber determines the important parameters of the M × N port WSS, such as the number of input/output ports and insertion loss. In this paper, VirtualLab Fusion software 2023.1 (Build 1.558), as a powerful physical optics simulation tool, is used to design and optimize a silicon micro-lens array that can achieve the high-precision coupling of a fiber array with a pitch of 1143 μm. Finally, the designed micro-lens is manufactured and experimentally demonstrates its good beam focusing ability with a 3 dB insertion loss. The designed micro-lens array coupling system, which delivers 28 focused spots of approximately 1mm in size (the beam has a $1/e^{2}$ diameter) after transmitting a distance of around 300 mm, effectively extends the number of WSS ports. This design method of the micro-lens array significantly amplifies the port count of the M × N port wavelength-selective switch, effectively expanding it to encompass an impressive 28 × 28 ports.

## 1. Introduction

The emergence of technologies such as the 5G access network, multimedia services, and artificial intelligence has accelerated the continuous growth in bandwidth demand in optical networks. The all-optical communication network based on ROADM (Reconfigurable Optical Add/Drop Multiplexer) technology has become the main direction for the current development of optical communication technology. The M × N port wavelength-selective switch (WSS), a crucial device used for Reconfigurable Optical Add/Drop Multiplexors and optical switching nodes in optical communication systems, continues to move towards higher switching capacity; thus, increasing the number of input/output ports in M × N port WSS has become one of the numerous research directions in the field of WSS.

**Figure 1:**
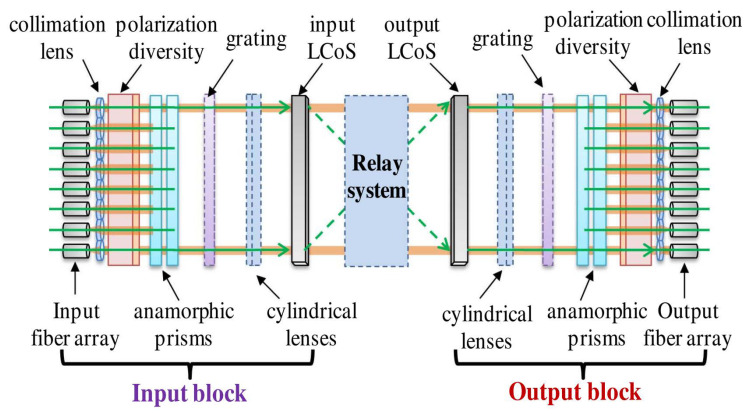
Schematic of WSS structure [1,2].

As shown in Figure 1, the main function of M × N port WSS is to convert the optical signal from a fiber to a spatial beam, and then, switch multiple beams to any target output ports [1,2,3]. During the design of this M × N port WSS, multiple parallel beams from M input ports transmit a long distance to reach the liquid crystal spatial light modulator (LC-SLM), with the light spots from each port non-overlapping in the LC-SLM. Since long transmission distances will lead to the serious dispersion of Gaussian beams, the most pressing challenge is determining how to design the micro-lens to ensure that the incident focused spot size remains as small as possible during free-space transmission while maximizing the number of focused spots within the constrained area of the LC-SLM [4]. Consequently, establishing the appropriate spot size becomes critical for expanding the number of WSS ports and has emerged as a vital research focus for advanced port WSS technology.

In order to enhance the transmission efficiency and performance of WSS, the mainstream WSS systems commonly employ fiber micro-lenses or larger-sized fiber collimators in their input/output modules [1,5,6]. In 2021, Qiang Hu conducted research on the MMA-WSS system, which utilized large-size fiber collimators for one input and twenty output ports. However, this strategy resulted in a reduction in available ports within the allocated space [7]. Similarly, in 2018, M. Iwama from the University of Cambridge employed a tapered waveguide array in a WSS, which suffered from high insertion loss and a structurally complex design involving deformed prisms, consequently limiting port availability [8]. As a resolution to this issue, they subsequently replaced the deformed prisms with micro-lenses, thereby enhancing system performance and expanding the number of ports [6,8]. This innovative approach effectively surmounted the challenges of limited port capacity and insertion loss, ultimately improving the overall efficiency of the WSS system. The fiber collimator array serves as another type of port array in WSS. However, dealing with a substantial number of ports presents a formidable challenge in adjusting the positioning and parallelism of each collimator after their dense arrangement, posing significant hurdles for mass production. In contrast, the fabrication processes applied to fiber arrays and micro-lens arrays offer a solution by ensuring precise positioning. Subsequent mature coupling processes further guarantee the parallelism of all port output beams. Consequently, fiber-coupling micro-lens is proven to be a promising means to augment the number of M × N port WSS ports because of the compact size of highly customizable micro-lenses.

In terms of the number of ports in the M × N WSS, Huawei employed two liquid-crystal chips to establish an 8 × 8 port WSS in 2014 [9]. Subsequently, in 2018, the company of Lumentum introduced an 8 × 24 port WSS [10], utilizing a combination of liquid crystal chips and Micro-Electro-Mechanical Systems (MEMS) for secondary beam deflection. Another development came in 2019 from Huazhong University of Science and Technology, reporting the creation of an 8 × 16 port WSS [11]. To address the challenge of densely arranged port outputs and ensure non-interfering parallel beams, the currently maximum reported output port quantity was limited to 24. This limitation arises primarily from the constraints imposed by micro-lens materials and size. In response to this, we introduce a silicon material port array, effectively increasing the number of ports to 28.

In this paper, we use a silicon design and demonstrate a micro-lens array with 28 ports that can maintain a beam diameter of 1mm over a beam transmission distance of 300 mm. The 3 dB coupling efficiency and 1 mm spot size of the designed micro-lens not only maximize the utilization of the receiving area, but also pave the way for the WSS to improve spatial light conversion capability and expand the number of ports. In contrast to the previously suggested designs, we innovatively expanded the port count of the M × N port WSS and employed high-refractive-index silicon materials in the production of micro-lens arrays. As a result, the experimental far-field beam size approached the theoretical transmission limit of Gaussian beams. This design method of the micro-lens array significantly amplifies the port count of the M × N port wavelength-selective switch, effectively expanding it to encompass an impressive 28 × 28 ports.

## 2. Beam Propagation Model

In the optical structure of M × N WSS, the core principle of the optical switching function is that multiple parallel beams from the M input ports are independently deflected by the LC-SLM to the N output ports, so we should ensure that the multiple Gaussian beams from the input ports remain non-overlapping in the LC-SLM. Meanwhile, the input/output ports should be closely aligned with each other to ensure the utilization of the liquid crystal chips and higher number of ports. According to the Gaussian beam transmission theory, a smaller beam waist leads to a larger divergence angle. Therefore, it becomes imperative to optimize the size and position of the Gaussian beam waist to arrange a greater number of ports within the limited available region of the LC-SLM. Oversized micro-lenses limit the number of ports, while undersized ones leads to beam divergence; striking the right balance ensures efficient spatial light conversion and maximizes the LC-SLM’s utilization, enhancing the system’s overall performance [5,6].

In our M × N WSS setup, the effective area of the 4K-resolution LC-SLM with a 3.74 μm pixel size measures 15.32 × 9.22 mm [3], and the designed micro-lens array is selected to transition the beam released from multiple fiber end faces into parallel and non-overlapping beams. The fiber array with a 127 μm pitch, readily available from the Planar Lightwave Circuit (PLC) optical splitter, serves as the input light signal port. The flexibility of choosing fibers with different spacing makes coupling with various micro-lens sizes convenient; thus, the dimensions of an individual micro-lens align with an integer multiple of 127 μm.

### 2.1. Characteristic of Gaussian Beam

The light beam projected from the fiber end face, which can reasonably be considered a Gaussian beam, undergoes conversion via a micro-lens and continues to conform to the Gaussian beam model [12]. As the transmission distance extends, the field intensity distribution of the Gaussian beam satisfies the following formula:

(1) $$u\left(x,y,z\right)=u_{0}\frac{\omega_{0}}{\omega (z)}\exp\left[-\frac{x^{2}+y^{2}}{\omega_{z}^{2}}\right]\exp\left[i\varphi \right]$$

In this equation, $u_{0}\frac{\omega_{0}}{\omega (z)}\exp\left[-\frac{x^{2}+y^{2}}{\omega_{z}^{2}}\right]$ represents the amplitude factor, $\exp\left[i\varphi \right]$ represents the phase factor, and ω(z) represents the beam radius at point ‘*z*’. The relationship can be described by the following equation:

(2) $$\omega \left(z\right)=\omega_{0}{\left[1+{\left(\frac{\lambda z}{\pi {\omega_{0}}^{2}}\right)}^{2}\right]}^{\frac{1}{2}}$$

**Figure 2:**
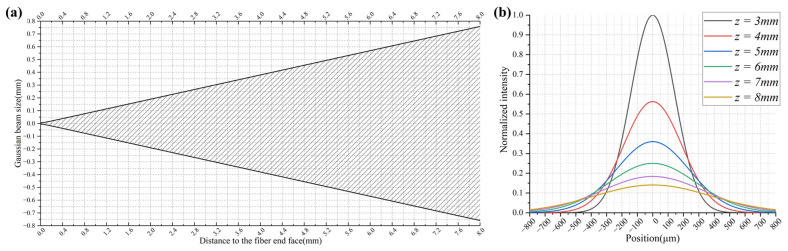
Schematic of fiber end beam transmission: (**a**) divergence of the Gaussian beam at the fiber end face; (**b**) different spot sizes of *z* = 3, 4, 5, 6, 7, 8 mm.

In this equation, $\omega_{0}$ represents the beam radius at the waist position, ‘*z*’ represents the distance from the waist position to any given position, and ‘λ’ denotes the wavelength of the beam. Considering that the mode field diameter of a single-mode fiber equals 10.4 μm, the corresponding radius of the 1550 nm Gaussian beam’s waist emitted from the fiber end face is 5.2 μm. By utilizing the amplitude distribution formula of the Gaussian beam, the size of the fiber spot at the micro-lens focal point can be determined. Figure 2 shows the divergence of the Gaussian beam at the fiber end face and the different spot sizes of *z* = 3, 4, 5, 6, 7, 8 mm.

**Figure 3:**
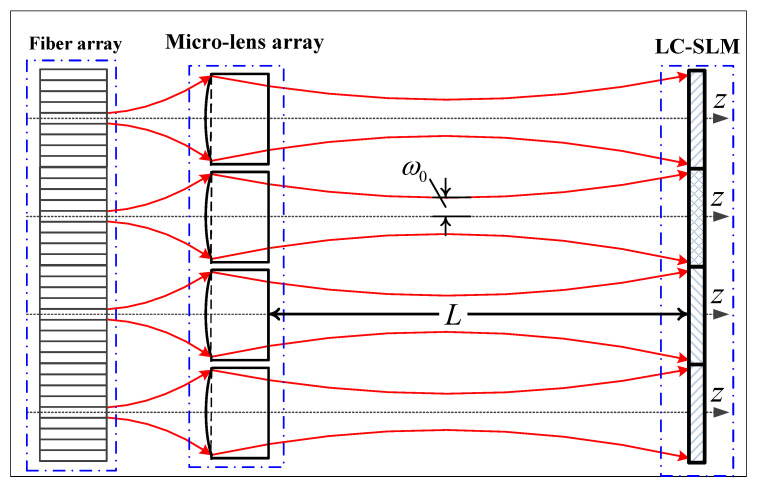
Beam schematic diagram of the fiber-coupling micro-lens. The red arrows indicate the direction of beam propagation.

In the design of the wavelength-selective switch, the fiber end should be positioned at the focal point of the micro-lenses to ensure coupling efficiency. Since the diffraction gratings, lenses, and other optical components located between the micro-lens and LC-SLM require approximately 300 mm of space, all Gaussian beams emitted from the micro-lens array need to transmit over 300 mm without overlapping, so the waist of the Gaussian beam emitted from the micro-lens is positioned midway between the LC-SLM plane and the micro-lens plane. The final beam schematic diagram of the fiber-coupling micro-lens is shown in Figure 3 [13]. The fiber array with a 127 μm pitch, readily available from the Planar Lightwave Circuit (PLC) optical splitter, is precisely arranged in a one-dimensional array. Owing to the utilization of spaced fibers in our system, the dimensions of each micro-lens are meticulously designed to be integer multiples of 127 μm, such as 635 μm, 762 μm, 889 μm, 1016 μm, and 1143 μm.

**Figure 4:**
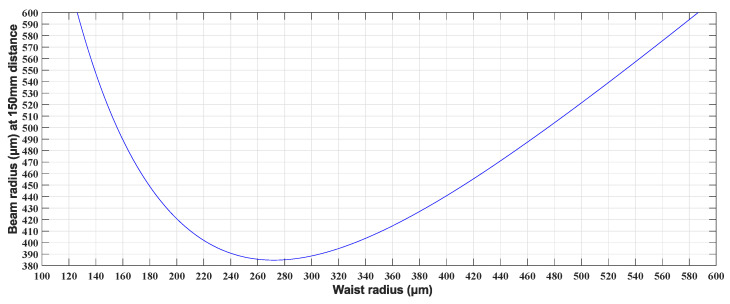
The relationship between waist radius and beam radius at a 150 mm distance.

The next step is to determine the range of the waist radius based on the transmission distance L=300 mm as shown in Figure 3, by using Equation (1). Due to the left–right symmetry of the Gaussian beam, the beam’s spot size in the LC-SLM plan at a transmission distance of 150 mm can be calculated from Equation (1) for a range of waist radii, as demonstrated in Figure 4. Thus, a Gaussian beam with a waist radius of approximately 280 μm is deemed fit for our WSS requirements.

Therefore, the objective of this paper is to determine the material, dimensions, and curvature radius of the micro-lens for converting a Gaussian beam with a 5.2 μm waist radius at the fiber end into a Gaussian beam with a 500 μm waist radius.

### 2.2. Beam Transmission Model

The single-fiber-coupling micro-lens system is shown in Figure 3, the emission of light from the fiber end face forms a waist with a Gaussian beam light field. The spot size and radius of curvature of the beam can be determined using the Gaussian beam formula [14]. When the Gaussian beam is transformed through the micro-lens, diffraction phenomena occur, significantly impacting light field calculations due to the small size of the beam spot at the fiber end. To attain precise optical field calculations in such situations, we turn to the Fresnel–Kirchhoff diffraction theory [15].

The Fresnel–Kirchhoff diffraction formula provides an efficient and accurate method to calculate the complex amplitude distribution and spot size after the micro-lens interaction [16,17]. The light field distribution of the LC-SLM plane at any position can be computed once we know the light field distribution after applying the micro-lens.

(3) $$U\left(P\right)=\frac{1}{j\lambda }{\;\int \int \;}_{S_{0}}U\left(Q\right)\left[\frac{\cos\left(n,r\right)-\cos\left(n,r_{0}\right)}{2}\right]\frac{e^{ikr}}{r}ds$$

To ensure the accuracy of the design calculations, the Gaussian light field passing through the micro-lens is divided into two parts: the lens surface and the lens substrate. This division is based on the structure of the micro-lens [18]. This design utilizes a plano-convex micro-lens, as depicted in Figure 4. This division allows for a comprehensive analysis of the light field’s behavior within the micro-lens structure, enabling precise optimization of the system’s performance [19].

**Figure 5:**
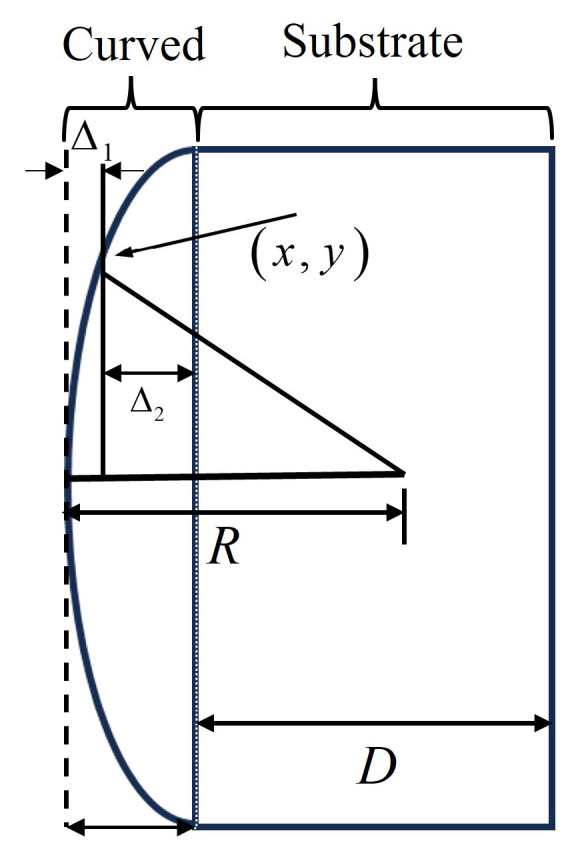
Micro-lens structure.

As shown in Figure 5, for the substrate part, the light can be considered to pass through a dielectric plate with refractive index *n* and thickness *D*. For the surface part, the thickness of the lens corresponding to any point (x,y) on the micro-lens consists of two parts:

(4) $$\begin{array}{c} \Delta_{1}=R-\sqrt{R^{2}-\left(x^{2}+y^{2}\right)}\\ \Delta_{2}=n\left[d-\left(R-\sqrt{R^{2}-\left(x^{2}+y^{2}\right)}\right)\right] \end{array}$$

The micro-lens surface thickness function is $D\left(x,y\right)=\Delta_{1}+\Delta_{2}$, and the phase modulation function can be expressed as follows: $t_{1}\left(x,y\right)=\exp\left[jkD(x,y)\right]$.

**Figure 6:**
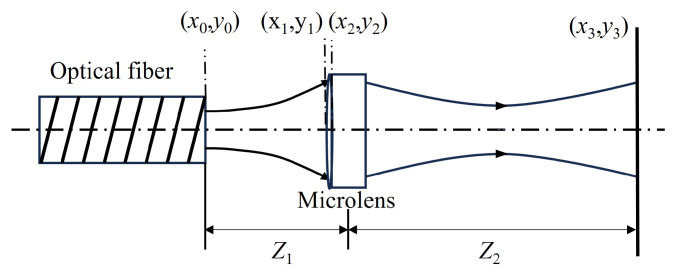
Transmission model of fiber-coupled micro-lens system.

According to $t_{1}\left(x,y\right)$, the phase of a Gaussian beam is modified when light waves pass through a micro-lens, while the amplitude remains unchanged. When the Gaussian beam exits the fiber port and reaches the lens, an equivalent optical path occurs, as illustrated in Figure 6. The propagation of the beam occurs along the *z*-axis, with the fiber end plane, the micro-lens front plane, the micro-lens back plane, and the receiving end plane designated as $\left(x_{0},y_{0}\right)$,$\left(x_{1},y_{1}\right)$,$\left(x_{2},y_{2}\right)$, and $\left(x_{3},y_{3}\right)$, respectively. The micro-lens focal length is denoted as $f_{1}$. According to the Gaussian beam, the light field distribution on the plane can be expressed as [20]:

(5) $$U_{1}\left(x_{1},y_{1}\right)=A_{0}\frac{\omega_{0}}{\omega \left(z\right)}\exp\left[-\frac{x_{1}^{2}+y_{1}^{2}}{\omega^{2}\left(z\right)}\right]\exp\left[i\left(-kz-k\frac{x_{0}^{2}+y_{0}^{2}}{2R(z)}+\arctan\left(\frac{z\lambda }{\pi \omega_{0}^{2}}\right)\right)\right]$$

where the spot size at distance *z* from the fiber end face is represented by $\omega \left(z\right)=\omega_{0}{\left[1+{\left(\frac{\lambda z}{\pi {\omega_{0}}^{2}}\right)}^{2}\right]}^{\frac{1}{2}}$, and the radius of curvature is $R\left(z\right)=z\left[1+{\left(\frac{\pi \omega_{0}^{2}}{\lambda z}\right)}^{2}\right]$. The field distribution $U_{2}\left(x_{2},y_{2}\right)$ on the $\left(x_{2},y_{2}\right)$ plane after the beam passes through the micro-lens is denoted as [21]:

(6) $$U_{2}\left(x_{2},y_{2}\right)=U_{1}\left(x_{1},y_{1}\right)t_{1}\left(x,y\right)$$

When the beam is transmitted in free space after leaving the micro-lens, the light field distribution in the receiving plane at a distance ‘*r*’ from the plane, according to Kirchhoff’s diffraction law, is given by

(7) $$\begin{array}{c} U_{3}\left(x_{3},y_{3}\right)=\frac{1}{j\lambda }{\;\int \int \;}_{\Sigma }U_{2}\left(x_{2},y_{2}\right)\left[\frac{1+\cos\theta }{2}\right]\frac{e^{ikr}}{r}ds\\ =\frac{1}{j\lambda }{\;\int \int \;}_{\Sigma }U_{1}\left(x_{1},y_{1}\right)t_{1}\left[\frac{1+\cos\theta }{2}\right]\frac{e^{ikr}}{r}dx_{1}dy_{1} \end{array}$$

The Gaussian beam field distribution formula and the Kirchhoff diffraction formula guide the light field distribution after applying the micro-lens to achieve approximate symmetry between the receiving end and the micro-lens plane around the position of the beam waist [22].

## 3. VirtualLab Fusion Software Simulation

VirtualLab Fusion 2023.1 (Build 1.558) is an optical modeling and simulation software platform developed by the German company LightTrans. Based on the concept of field tracing, it provides a wealth of features and toolboxes for tracing light rays and fields, modeling and analyzing optical elements, optimization, and designing diffractive optical elements. The simulation and optimization of the fiber-coupling micro-lens system are carried out with VirtualLab Fusion optical software, leading to the determination of the micro-lens’ radius of curvature and dimensions.

For the simulation of the mode field diameter of the single-mode fiber’s end face, the light source is configured as a Gaussian beam with a wavelength of 1550 nm and a beam waist radius of 5.2 μm. The following step requires the configuration of micro-lens parameters, including important considerations such as material selection, focal length, and radius of curvature.

**Figure 7:**
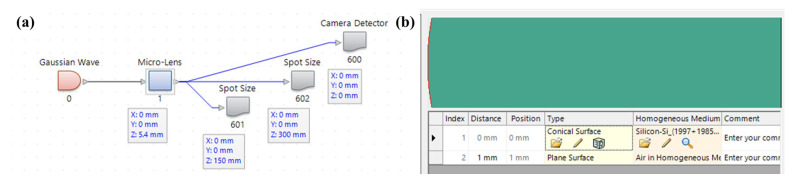
(**a**) Schematic block diagram of simulation components (**b**) Micro-lens structure setup interface.

Micro-lenses are constructed using two widely used materials, fused silica and high-purity silicon. Subsequently, the radius of curvature is optimized to guarantee high coupling efficiency and a collimated beam in simulations. The Gaussian beam amplitude distribution formula demonstrates that the $1/e^{2}$ spot size grows to 1mm when the beam is conveyed 5.47 mm from the fiber end face. The optical path structure and micro-lens structure setup interface as illustrated in Figure 7 is constructed, whereby the initial focal length of the micro-lens is set to 5.47 mm and the energy cutoff position of the spot size detector is set to $1/e^{2}$.

Regarding optimization of the longitudinal position of the optical fiber, when the optical fiber couples with the micro-lens array as the output port of the beam, since the optical fiber needs to receive the focused beam of the micro-lens, the position of the optical fiber is always located along the focal length of the micro-lens. The spot sizes of micro-lenses fabricated from fused silica and silicon were contrasted at distances of 150 mm and 300 mm under varying focal lengths. The focal length optimization range extends from 1 mm to 15 mm, with a step size of 560 μm.

**Figure 8:**
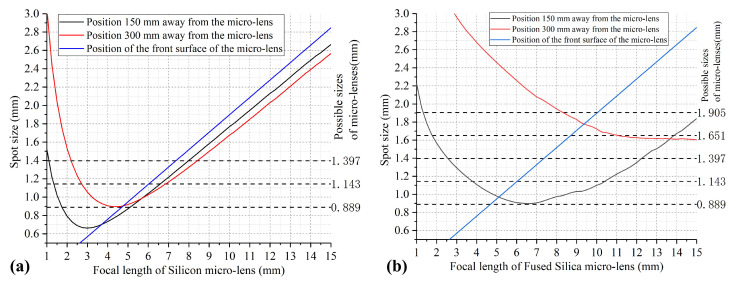
Variation in focused spot size with different focal lengths of fused silica and silicon: (**a**) Spot size with different focal lengths of fused silica micro-lens (**b**) Spot size with different focal lengths of silicon micro-lens.

The simulation results in Figure 8 illustrate the beam transmission size for micro-lenses of varying focal lengths and materials. The fluctuation observed in the curve arises from positioning errors encountered while placing the fiber along the micro-lens’ focal length during each calculation. However, it does not significantly impact the overall trend depicted by the curve.

The focal length of the micro-lens determines the distance between the fiber and the micro-lens, as well as the size of the spot where the output beam reaches the lens. A larger focal length results in a larger spot size, and excessively large spots may hinder an increase in the number of ports. Based on the optimization results of Figure 8, when the silicon micro-lens has a size of 1.143 mm and a focal length of approximately 5 mm, the beam’s spot size remains below 1mm throughout the transmission process. In contrast, the fused silica micro-lens must have a minimum $1/e^{2}$ size of 1.905 mm to attain a beam size smaller than 1.9 mm. Consequently, we opt for a silicon material to fabricate micro-lenses capable of achieving smaller beam sizes; prioritizing silicon micro-lenses in our experimental design is anticipated to yield superior performance in the context of the M × N port wavelength-selective switch.

## 4. Experiment Verification

The micro-lens array using the silicon material was fabricated by using the chemical etching method. Each individual lens has a curvature radius of 13 mm, focal length of 5.25 mm, and dimensions of 1.13 mm, the center-to-center distance between adjacent lenses is 1.143 mm. To enhance its performance, the micro-lens array was coated with an anti-reflective film specifically designed for a wavelength of 1550 nm.

**Figure 9:**
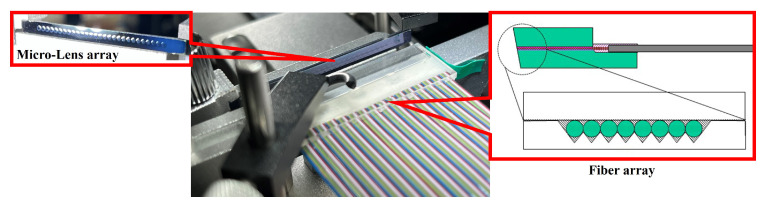
Schematic diagram of fiber array and micro-lens array.

The fiber arrays utilized a well-established PLC optical splitter production process, employing V-groove fixation technology [4,6]. The single-mode fiber spacing was set at 127 μm. The fiber array is shown in Figure 9. To match the completed micro-lens array with 28 ports, we customized a fiber array with 256 fibers, where each set of 9 fibers corresponds to one micro-lens.

**Figure 10:**
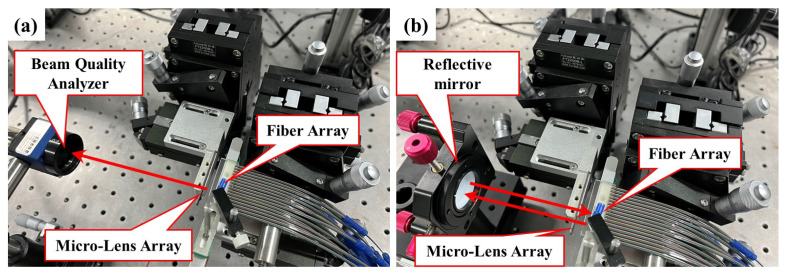
(**a**) System setup of fiber-coupling micro-lens. (**b**) Measurement of coupling efficiency of fiber-coupling micro-lens arrays.The red arrow represents the direction of the beam.

As shown in Figure 10a, a high-precision six-dimensional adjustment platform was used for the accurate alignment of fibers with the micro-lens. In the experimental process of aligning the fibers with the micro-lens, the first step was to adjust the spacing between the fibers and the micro-lens to minimize loss, and then, adjust the rotation angle of the micro-lens. The position of each port was adjusted appropriately based on the beam quality analyzer imaging to achieve optimal alignment for all ports.

**Figure 11:**
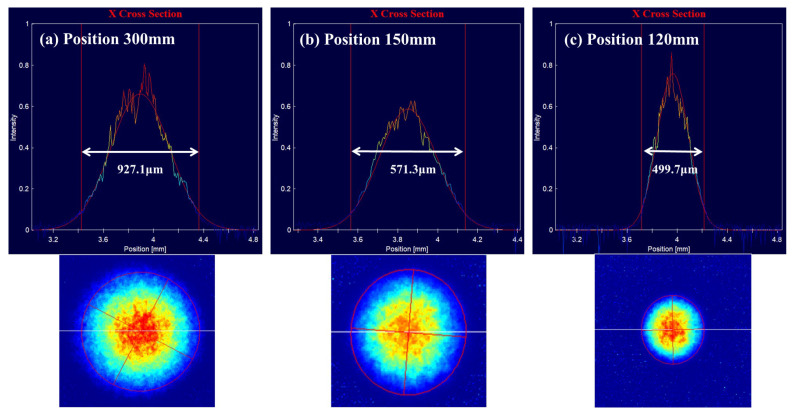
Measurement of spot images at different distances away from the micro-lens. The images captured by the beam quality analyzer feature red circles, symbolizing the $1/e^{2}$ spot sizes. The one-dimensional energy curve above is constructed by measuring the light intensity at the position of the white line.

After aligning the micro-lens with the optical fiber, the $1/e^{2}$ spot sizes were measured at positions of 120 mm, 150 mm, and 300 mm from the micro-lens, as shown in Figure 11. It can be seen that the spot size at the $1/e^{2}$ position at 300 mm is less than 1 mm.

**Figure 12:**
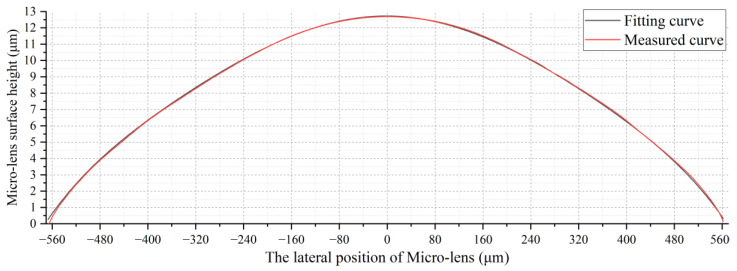
Micro-lens surface measurement and fitting curve.

Through the above experiments, it can be observed that the $1/e^{2}$ spot size of the beam at different positions deviates slightly from the simulated calculation results in Figure 8. The main reason for this is that the chemical etching method resulted in a micro-lens surface type inconsistent with our design. The micro-lens surface type curve obtained through the step profiler test is shown in Figure 12, and it is clearly noticeable that the curve is somewhat conical. Therefore, we continued to use VirtualLab Fusion software to fit this surface type using the VirtualLab aspherical surface curve formula [23]:

(8) $$\begin{array}{c} h\left(x,y\right)=\frac{cr^{2}}{1+\sqrt{1-\left(1+\kappa \right)c^{2}r^{2}}}\\ c=\frac{1}{R}\;r=\sqrt{x^{2}+y^{2}} \end{array}$$

By continuously optimizing the radius of curvature *R* and conical constant *k* in the above formula using genetic algorithms, a fitted curve was obtained, where *R* = 11.514 mm and *k* = −316.47. The fitting curve and the measured curve are shown in Figure 12. When fixing the radius of curvature *R* and experimenting with different conical constant *k* values, we observed that *k* primarily influences the surfaces of the micro-lenses at positions −550∼−300 μm and 300∼550 μm. As the main energy of the Gaussian beam precisely concentrates at the center position of the micro-lens, it is crucial to maintain strict consistency in the radius of curvature *R* during the micro-lens manufacturing process. The restriction on the conical constant *k* can be moderately relaxed. Therefore, when using global optimization algorithms such as genetic algorithms for curve fitting, weighting factors can be added to both *R* and *k*. Giving significantly more weight to *R* compared to *k* ensures that the fitting results of the lens surface are closer to reality.

**Figure 13:**
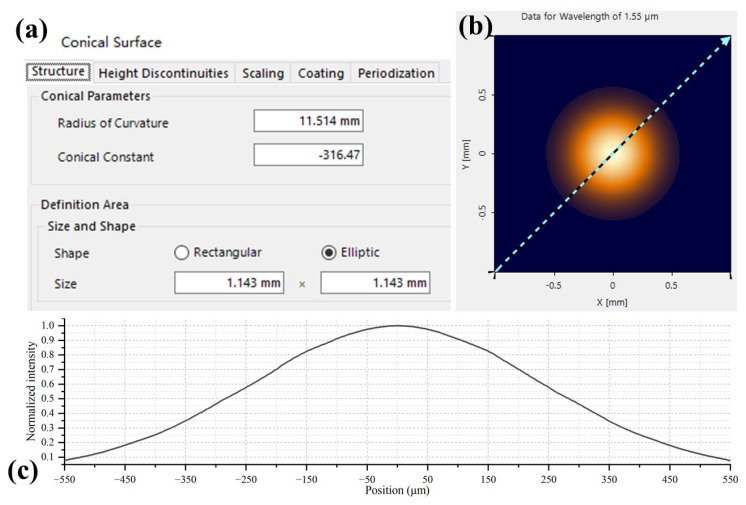
(**a**) Micro-lens setting in VirtualLab Fusion. (**b**) Spot image at a position of 300 mm away from the micro-lens. (**c**) Normalized intensity curve of spot cross section.

**Figure 14:**
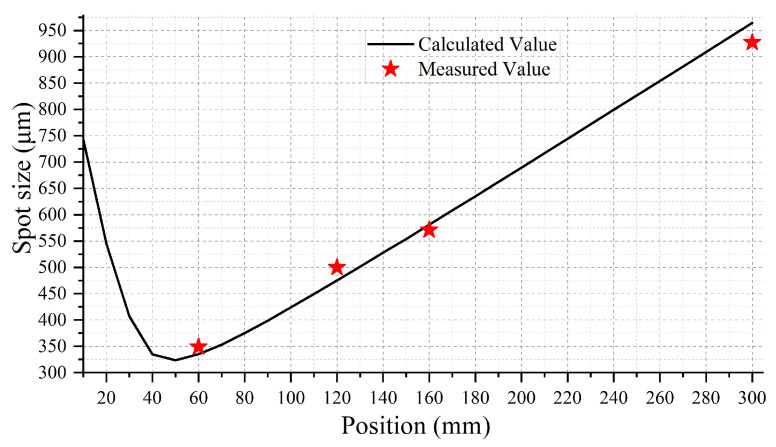
Comparison of measured and calculated $1/e^{2}$ spot sizes at different distances.

Using the previous simulation system settings, only the micro-lens surface type was modified in Figure 13. The simulation outcomes are presented in Figure 13 and Figure 14. Remarkably, in Figure 14, the experimental results align closely with the calculated values. The beam waist is located about 50 mm from the micro-lens, with a beam waist radius of 251 μm. The beam’s $1/e^{2}$ size remains below 1mm within a 300 mm range from the micro-lens. These findings affirm that the micro-lens parameters validate its appropriateness for producing collimated beam arrays.

In a WSS, the beam deflected by the LC-SLM needs to be coupled back into the optical fiber. Next, we used reflective mirrors instead of the LC-SLM to test the coupling efficiency of the micro-lens. As shown in Figure 10b, upon adjusting the position of the reflective mirror to optimize the coupling efficiency, the experiments reveal that positioning the reflective mirror 60 mm from the lens achieves a highest coupling efficiency, and the beam’s energy reflected back to the fiber is reduced to 50% of that of the outgoing beam. It can be seen from Figure 14 that the main reason for this energy loss is that the reflected light cannot remain consistent with the outgoing light from the fiber end face. The total loss of the fiber-coupling micro-lens system is measured at an optimal value of 3 dB.

**Figure 15:**
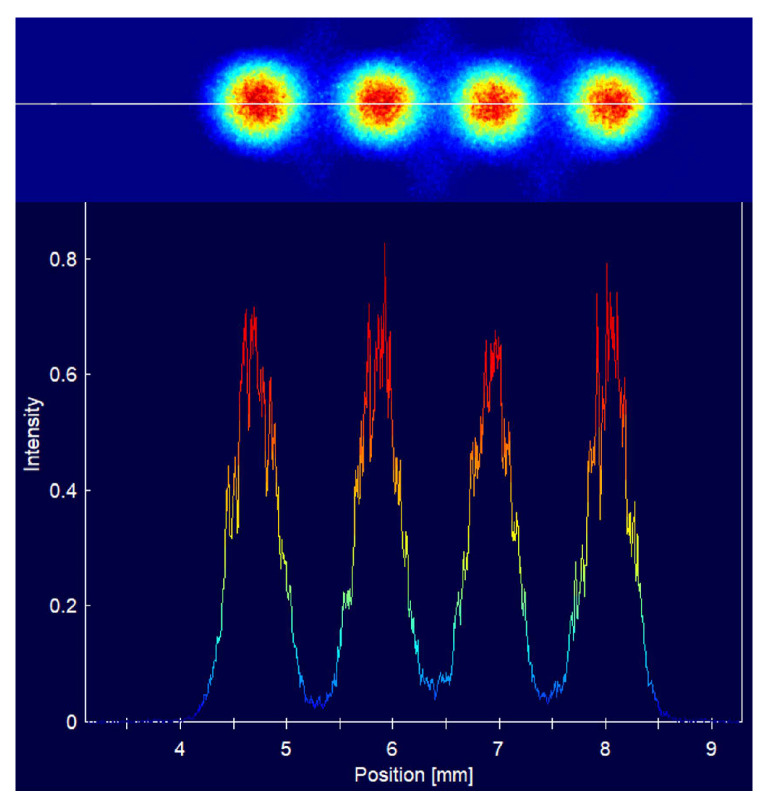
Images of multiple spots at the 300 mm position. The one-dimensional energy curve below is plotted by measuring the light intensity at the position of the white line on the beam quality analyzer image above.

Finally, we tested a situation where multiple ports simultaneously output light signals at a distance of 300 mm from the micro-lens, as shown in Figure 15.

**Figure 16:**
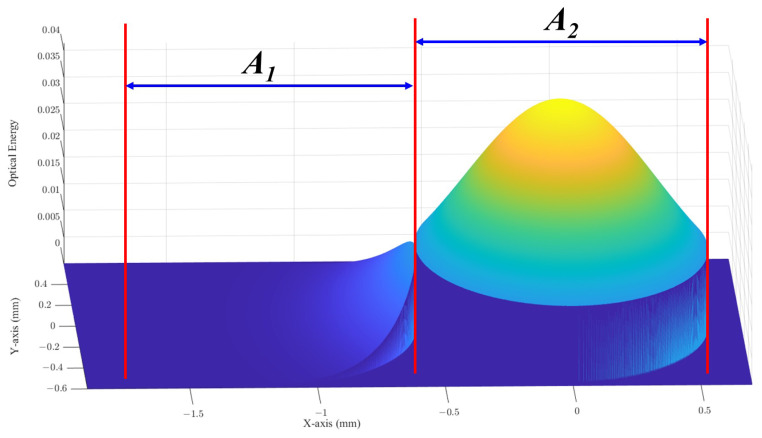
Images of port crosstalk.

As shown in Figure 16, port crosstalk is defined as the proportion of light entering the adjacent port area; thus, crosstalk can be assessed by examining the light distribution in the neighboring port region [24,25,26]. It is known that the waist radius of the Gaussian beam behind the micro-lens is 165 μm, and the Gaussian beam field distribution at the 300 mm position can be calculated based on Equation (5). Then, the optical power within the two port areas can be given by

(9) $$P_{1}=\underset{A_{1}}{\;\int \int \;}{\left|U_{1}\left(x_{1},y_{1}\right)\right|}^{2}dx_{1}dy_{1}$$

(10) $$P_{2}=\underset{A_{2}}{\;\int \int \;}{\left|U_{1}\left(x_{1},y_{1}\right)\right|}^{2}dx_{1}dy_{1}$$

$A_{1}$ represents the areas covered by one of the neighboring ports. $A_{2}$ is the area covered by the intended port.

The crosstalk ratio *R* (dB) can be determined as

(11) $$R=10\log_{10}\frac{P_{1}}{P_{2}}$$

**Figure 17:**
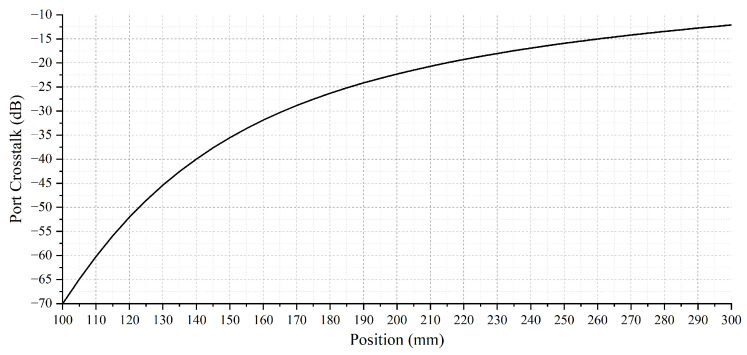
The port crosstalk R at different positions.

Based on the calculation, the port crosstalk *R* in this experiment at different positions is shown in Figure 17. Typically, in optical communications, it is necessary to reduce the port crosstalk to below −20 dB; thus, it is also necessary to appropriately shorten the optical beam transmission distance based on the port crosstalk situation.

The designed fiber array and the silicon micro-lens enable precise coupling of the optical signals, ensuring efficient and reliable optical transmission and exchange. The experimental results are in congruence with the theoretical design, confirming the feasibility and effectiveness of the fiber-coupled micro-lens system for wavelength-selective switch application. However, although the beam size meets the requirements, the coupling efficiency still needs to be improved.

## 5. Conclusions

To augment the port count of the M × N port wavelength-selective switch (WSS) and enhance overall system performance, a fiber-coupling silicon-based micro-lens array is proposed. We used the VirtualLab Fusion software for comprehensive design simulation and optimization. An optical fiber-coupling silicon micro-lens array with 28 ports was designed and fabricated. However, owing to errors in the chemical etching method, surface errors in the actual micro-lenses led to coupling losses. The accuracy of the experimental results was validated using Virtuallab simulations, confirming a measured loss of 3 dB for the fiber-coupling micro-lens array. The next step involved further optimizing the micro-lenses to reduce their coupling losses while simultaneously enhancing the beam quality. This design method of the micro-lens array significantly amplifies the port count of the M × N port wavelength-selective switch, effectively expanding it to encompass an impressive 28 × 28 ports. This achievement holds paramount significance in the context of constructing future all-optical switching nodes.

## Data Availability

Data are contained within the article.
